# La mucormycose rhino-orbito-cérébrale

**Published:** 2012-11-29

**Authors:** Ali Derkaoui, Mohammed Khatouf

**Affiliations:** 1 Service d’anesthésie réanimation A1 CHU HASSAN II Fes, Maroc

**Keywords:** Mucormycose, diabète, amphotericine B, mortalité, mucormycosis, diabetes, amphotericin B, mortality

## Image en médecine

La mucormycose est une infection fongique rarissime et souvent fatale, elle est due à la prolifération dans différents tissus de champignon opportuniste de la famille des mucorales. Cette infection survient chez les patients immunodéprimés, notamment, les diabétiques décompensés. L’atteinte rhino-orbito-cérébrale représente la moitié de toutes les formes cliniques, mettant en jeu le pronostic fonctionnel puis vital. Les auteurs rapportent l’observation d’un patient diabétique ayant présenté des lésions nécrotiques extensives de l’hémiface droite, non améliorées par un traitement antibiotique à visée antistaphylococcique. La TDM cérébro-faciale a objectivé un comblement du sinus sphénoïdalet des cellules ethmoïdales, avec épaississement et infiltration des parties molles préseptales et jugales droites Les examens anatomopathologique ont permis de poser le diagnostic de mucormycose. L’évolution a été fatale, malgré un traitement médico-chirurgical associant Amphotéricine B et débridement chirurgical.

**Figure 1:**
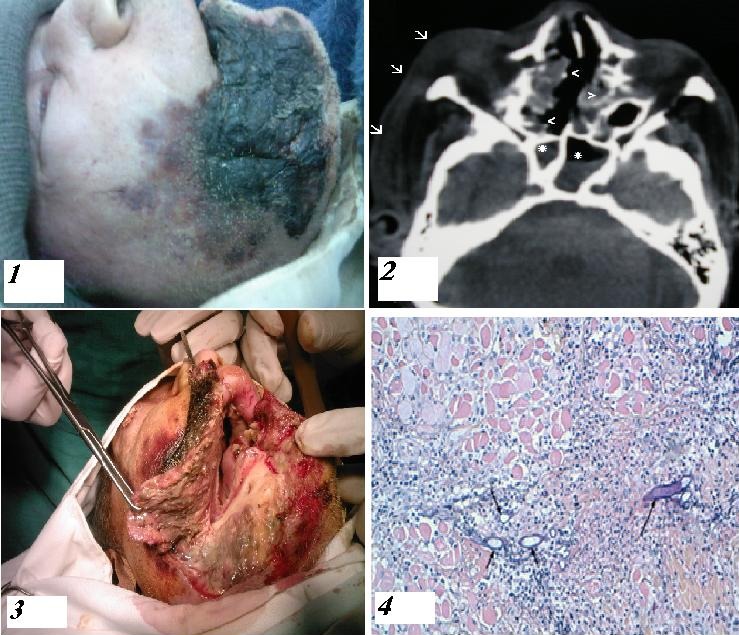
(1) Large nécrose tégumentaire jugale droite étendue aux régions labiale inférieure, mentonnière, et sous maxillaire homolatérales; (2): Coupe scanographique passant par le plan orbito-méatal montrant un comblement du sinus sphénoïdal (astérisque) et des cellules ethmoïdales (têtes de flèches), avec épaississement et infiltration des parties molles préseptales et jugales droites (flèches); (3): vue per opératoire prise lors de la nécrosectomie et du débridement chirurgical; (4): Coupes anatomopathologiques montrant des coulées de PNN avec des filaments mycéliens (flèches)

